# My Own Experience!

**Published:** 1859-12

**Authors:** 


Throw physic to the Dogs, I'll none of It.—*Shakspeare.*

### MY OWN EXPERIENCE!

*Or how to get rid of a Cold and its attendent ails and aches, viz., rheumatic pains, head ache, depression of spirits, etc.*

BY A BACHELOR.

---

Take an hour's walk, *regardless of the weather*, immediately before retiring to rest at night. Put on an overcoat, or *two* if necessary, and overshoes also, if the dampness of the weather requires it. With *mouth closed*, head erect, and with the shoulders thrown back, to enable you to breathe the more freely, walk half an hour from home, and the other half back. Avoid all "*Lager Bier Saloons*," or drinking places, *Oyster Houses* or *Restaurants*, on the way. Get into bed at once upon reaching home; cover up warmly, and my word for it, you will be quite clear of your cold *if a recent one*, the next morning, besides having had a good night's rest. But should the *cold* still continue, repeat the walk the following evening, *observing the same precautions*, and the next evening if necessary, If you have a sore throat or a tooth-ache, wrap a tippet around your throat and the lower part of your face in addition to your other covering, and keep the tippet on during the night, taking it off in the morning, but, if need be, keep it on during the following day, but do not take it off until you go to bed at night, or until the following morning.

Having given the foregoing a test, for a series of years, and having thereby warded off all the serious results which *inevitably follow* neglected colds, I have saved myself the necessity of taking medicine of any description, and from detention from business.

N. B.—Night air is said to be *unhealthy*. So it is, but you will experience no ill effects from it, so long as you *keep in motion*, and do not stop by the way!

☞ *The experience of one who may be met, any evening, on Broadway—stormy evenings not excepted—between the hours of* eight and nine o'clock, *taking his accustomed hour's walk, before retiring to rest.*

